# More and more pleiotropy within the IL‐6 family of cytokines

**DOI:** 10.1111/febs.17355

**Published:** 2024-12-13

**Authors:** Stefan Rose‐John, Simon A. Jones

**Affiliations:** ^1^ Department of Biochemistry Christian‐Albrechts‐University Medical School Kiel Germany; ^2^ Department of Infection, Immunity and Biochemistry, The School of Medicine Cardiff University UK

**Keywords:** binding specificity, cytokine receptors, cytokines, gp130, interleukin‐6 family, pleiotropy

## Abstract

Historically, cytokines belonging to the gp130 family bind to specific ligand‐binding receptors that stimulate cell signaling through a receptor complex comprising gp130 or gp130 together with another structurally related signaling receptor. However, recent findings increasingly dispel these stereotypes and suggest that the receptor specificity of gp130‐activating cytokines is less strict than originally assumed. Weitz *et al*. now provide the latest example of this pleiotropy and report that human interleukin‐6 can bind and stimulate signaling via the interleukin‐11 receptor. Possible biological and therapeutic consequences of these findings are discussed.

## Graphical abstract

Comment on: https://doi.org/10.1111/febs.17309.

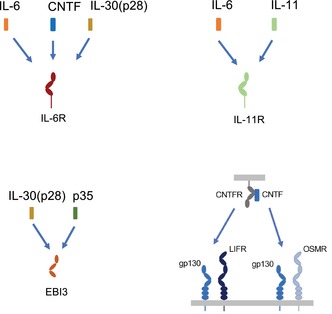

**Abbreviations**
CLC: cardiotrophin‐like cytokine
CNTF: ciliary neurotrophic factor
CNTFR: CNTF receptor
CT‐1: cardiotrophin‐1
EBI3: Epstein–Barr virus‐induced gene 3 (cytokine receptor‐like subunit of IL‐27 or IL‐35)
ERK: extracellular signal‐regulated kinase
gp130: glycoprotein 130 kDa
GPL: gp130‐like (signaling subunit of the IL‐31 receptor)
IL: interleukin
LIF: leukemia inhibitory factor
LIFR: LIF receptor
MAPK: mitogen‐activated kinase
OSM: oncostatin M
OSMR: OSM receptor
p35: cytokine‐like subunit of IL‐12

## Introduction

**Fig. 1:**
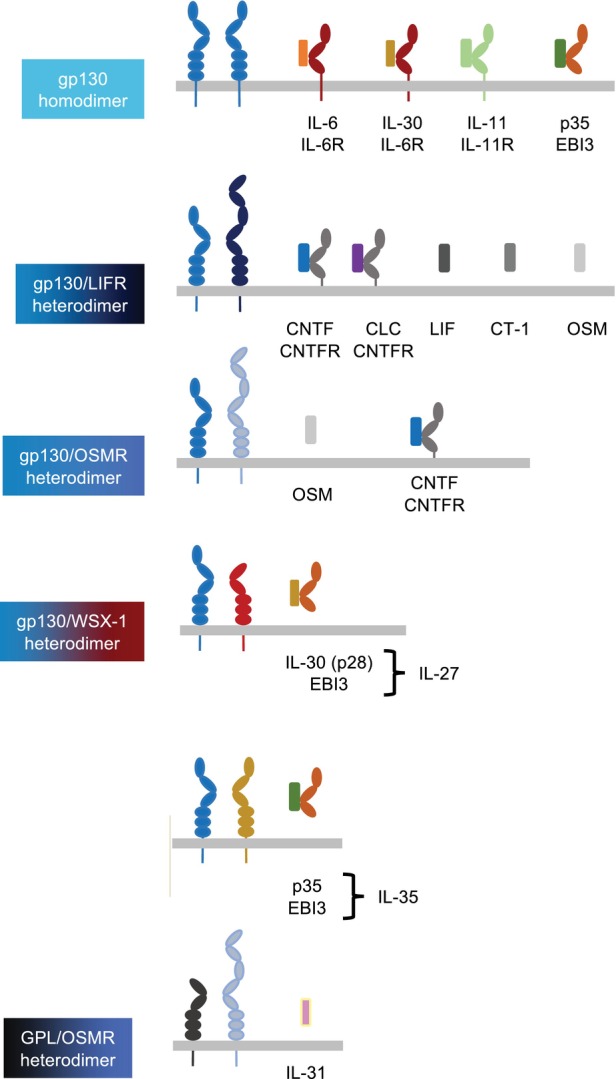
Overview of the IL‐6 family of cytokines. Homodimers of gp130 and heterodimers of gp130 with related receptors are shown along with their cognate cytokines or cytokine/receptor complexes. Details are given in the text.

**Fig. 2:**
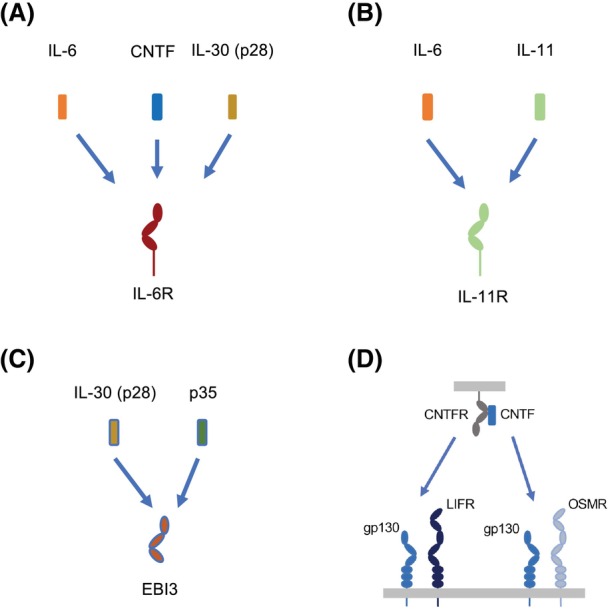
Pleiotropy of some cytokines or cytokine receptors of the gp130 family. (A) The IL‐6R can interact with the cytokines IL‐6, CNTF and IL‐30 and induce signaling via a gp130/LIFR heterodimer in the case of CNTF and via a gp130 homodimer in the case of IL‐30. (B) The IL‐11R can bind IL‐11 and IL‐6 and induce signaling via a gp130 homodimer. (C) The cytokine receptor EBI3 can interact with IL‐30 and with p35, which is a cytokine‐like subunit of the cytokine IL‐12. (D) The complex of CNTF and CNTFR can interact and signal via a heterodimer of gp130 and LIFR and via a heterodimer of gp130 and OSMR.

The four‐helical cytokines involved in coordinating the immune system are grouped into families defined by their common use of shared receptor subunits. Accordingly, members of the gp130 cytokine family use gp130 homo‐ or heterodimers as signaling receptors. These cytokines engage specific ligand‐binding receptors (IL‐6R, IL‐11R, EBI3, CNTFR) that establish signaling complexes through binding gp130 homo‐ or heterodimers (Fig. 1) [1]. Interestingly, the specific receptors also function as soluble proteins that complex with their ligands to stimulate gp130 signaling on cells lacking expression of their membrane‐bound counterparts. This mechanism, called trans‐signaling, dramatically expands the spectrum of target cells since gp130 and the gp130‐related receptors leukemia inhibitory factor receptor (LIFR) and oncostatin M receptor (OSMR) are far more widely expressed than the ligand‐binding receptors [2]. However, the specificity of these interactions for particular cytokines within the gp130 cytokine family is becoming more blurred. For example, it was already known that the interleukin (IL)‐6 receptor (IL‐6R) can bind ciliary neurotrophic factor (CNTF) and IL‐30 (I‐L27p28) and that Epstein–Barr virus‐induced gene 3 (EBI3) can bind IL‐30 (forming I‐L27) and p35 (forming I‐L35) [3], indicating some degree of pleiotropy. Furthermore, it was shown that CNTF can also activate the heterodimer of gp130 and OSMR (Fig. 2) [4].

Weitz *et al*. [5] now demonstrate that human IL‐6 can also bind the human IL‐11R mimicking signaling of IL‐11 via a gp130 homodimer (Fig. 2). Interestingly, this also applies to a chimeric engineered cytokine called IC7, which contains a binding site to IL‐6R, gp130, and LIFR and which has shown promising results in the treatment of type 2 diabetes and muscle atrophy [6, 7]. Remarkably, murine IL‐6 did not stimulate the murine IL‐11R/gp130 complex and IL‐11 did not cross‐react with IL‐6R [5].

Members of the IL‐6 cytokine family control a wide range of overlapping activities. This level of redundancy and pleiotropy is often explained by their universal reliance on gp130 for cell signaling and the ubiquitous cellular expression of gp130. In contrast, cognate receptors for individual family members display a more restricted expression and often correspond with the biological functions assigned to a gp130‐activating cytokine. As a result, the phenotype of mice genetically lacking a gp130‐activating cytokine (or their cognate ligand‐binding receptor subunit) is less dramatic than that of gp130‐deficient mice, which are embryonically lethal. While these studies help to compartmentalize the functional properties of members of the IL‐6 cytokine family, the ability of these cytokines to engage different receptor systems now raises questions for the field.

## Does this toggling of receptor usage have biological relevance?

Historical data show that certain gp130‐activating cytokines have evolved strategies that broaden their biological impact. For example, trans‐signaling mechanisms that broaden the types of cells that respond to IL‐6, IL‐11, and CNTF. However, the ability of gp130‐activating cytokines to share ligand‐binding receptor subunits, as described by Weitz *et al*., adds another layer of regulation. Earlier studies of OSM signaling provide an example of this complexity. Human OSM can signal through gp130 receptor cassettes comprising either the OSMR or the LIFR, and when signaling through LIFR, OSM can control activities commonly associated with LIF (e.g., hematopoiesis, totipotency). Equally, CNTF binds with low affinity to the IL‐6R and may elicit IL‐6‐like activities within the central nervous system through a gp130 receptor cassette comprising IL‐6R and LIFR (Fig. 2). It would be important to consider why these shared relationships exist. Do these interactions yield differences in signaling potentials, as illustrated by studies describing the activation of extracellular signal‐regulated kinase/mitogen‐activated protein kinase (ERK/MAPK) signaling by IL‐11, which has been claimed to be more prominent than that reported for IL‐6 [8]. Reflecting on the data from Weitz *et al*., *Il6ra* ^−/−^ mice have been shown to display heightened ERK/MAPK signaling during wound healing [9]. If indeed IL‐6 signaling and IL‐11 signaling is different, it will be interesting to molecularly define the signal transduction pathway when IL‐6 acts via the IL‐11R.

## Should we now reconsider the mode of action of biological medicines targeting IL‐6?

The potential for IL‐6 to engage and signal via the IL‐11R, as demonstrated by Weitz *et al*., now raises questions about the biological significance of this interaction and potentially means that biological medicines targeting IL‐6 itself (e.g., siltuximab, clazakizumab, and olokizumab) may possess different modes of action than IL‐6R blocking therapies (e.g., sarilumab, tocilizumab) (Fig. 2).

This might be important for several reasons. In mice, IL‐11 was recently shown to promote chronic inflammation in fibrotic lung disease and liver fibrosis [8]. Consequently, antibodies targeting IL‐11 or the IL‐11R are in clinical trials as drug candidates for treating lung fibrosis and nonalcoholic steatohepatitis [8]. Furthermore, it was most recently observed in a mouse model that pharmacologic and genetic inhibition of IL‐11 extended the health and lifespan of the animals, indicating that blocking IL‐11 could be a strategy for treating elderly human patients [10]. In this regard, it would be important to consider that in humans, IL‐6 could substitute for IL‐11, circumventing the pharmacologic blockade. As a note of caution, this effect would not have been observed in animals since murine IL‐6 does not cross‐react with the murine IL‐11 receptor [5].

In this regard, *Il6* ^−/−^ and *Il6ra* ^−/−^ mice show marked differences in wound healing, colitis severity and glucose metabolism, whereas comparisons between *Il11* ^−/−^ and *Il11ra* ^−/−^ mice display phenotypic differences in bone deformities (e.g., craniosynostosis seen in *Il11ra* ^*−/−*^ mice) and pulmonary fibrosis, which is protected in *Il11* ^−/−^ mice [9, 11]. However, in this case, the binding of IL‐6 to the IL‐11R cannot be responsible since murine IL‐6 does not signal via the murine IL‐11R [5]. This might point to even more pleiotropy within the gp130 cytokine family.

Still, the findings offered by Weitz *et al*. need to be placed in the context of clinical trials with tocilizumab, which has typically failed in indications where IL‐6 contributes to epithelial homeostasis, barrier integrity or immunity against infection [12]. For example, would interventions that target inflammatory flares in IL‐6 during chronic disease offer an advantage over a global blockade of IL‐6R, which may underpin physiological processes required for health?

## How does the sharing of receptor usage affect our understanding of IL‐6 and IL‐11 regulation?

Traditionally, the assignment of biological functions to IL‐6 and IL‐11 was broadly separated by the action of IL‐6 on leukocytes and IL‐11 on non‐hematopoietic stromal cells. Over time, this distinction has blurred with the discovery that sgp130 blocks both IL‐6 and IL‐11 trans‐signaling, illustrating a more common biological regulation. So, how should we compartmentalize the activities of IL‐6 and IL‐11 and contextualize the importance of trans‐signaling mechanisms if IL‐6 can engage other gp130‐related receptor systems? When considering these questions, it is important to reflect on the roles these cytokines play in healthy physiology versus those involved in pathophysiology and examine how the biological functions of IL‐6 and IL‐11 align. For example, studies of the musculoskeletal system show that IL‐6 may shape skeletal muscle function and regeneration, whereas IL‐11 may take a more central role in regulating bone turnover [13, 14]. A similar scenario exists in gastric cancer, where IL‐6 controls activities linked to angiogenesis, cancer inflammation and tissue remodeling, and IL‐11 instructs cancer cell survival and proliferation [15].

## Conclusion

In summary, the plot thickens, with Weitz *et al*. offering new light through old windows. These data provide provocative insights into the workings of IL‐6 and IL‐11 by offering a new understanding of how cells sense cytokine cues. The challenge for the research community is to establish how these types of receptor interactions affect the interpretation of these cytokine cues.

## Conflict of interest

The authors declare no conflict of interest.

## Author contributions

SR‐J and SAJ wrote and revised the manuscript together; SR‐J prepared the figures.

## References

[1] Garbers C , Heink S , Korn T & Rose‐John S (2018) Interleukin‐6: designing specific therapeutics for a complex cytokine. Nat Rev Drug Discov 17, 395–412. 29725131 10.1038/nrd.2018.45

[2] Rose‐John S & Heinrich PC (1994) Soluble receptors for cytokines and growth factors: generation and biological function. Biochem J 300, 281–290. 8002928 10.1042/bj3000281 PMC1138158

[3] Garbers C , Hermanns H , Schaper F , Müller‐Newen G , Grötzinger J , Rose‐ John S & Scheller J (2012) Plasticity and cross‐talk of interleukin 6‐type cytokines. Cytokine Growth Factor Rev 23, 85–182. 22595692 10.1016/j.cytogfr.2012.04.001

[4] Rafii P , Cruz PR , Ettich J , Seibel C , Padrini G , Wittich C , Lang A , Petzsch P , Kohrer K , Moll JM *et al*. (2024) Engineered interleukin‐6‐derived cytokines recruit artificial receptor complexes and disclose CNTF signaling via the OSMR. J Biol Chem 300, 107251. 38569939 10.1016/j.jbc.2024.107251 PMC11039321

[5] Weitz HT , Ettich J , Rafii P , Wittich C , Schultz L , Frank NC , Heise D , Krusche M , Lokau J , Garbers C *et al*. (2024) Interleukin‐11 receptor is an alternative α‐receptor for Interleukin‐6 and the chimeric cytokine IC7. FEBS J. In press. doi: 10.1111/febs.17309 PMC11796321 39473075

[6] Kallen KJ , Grotzinger J , Lelievre E , Vollmer P , Aasland D , Renne C , Mullberg J , Myer zum Buschenfelde KH , Gascan H & Rose‐John S (1999) Receptor recognition sites of cytokines are organized as exchangeable modules. Transfer of the leukemia inhibitory factor receptor‐binding site from ciliary neurotrophic factor to interleukin‐6. J Biol Chem 274, 11859–11867. 10207005 10.1074/jbc.274.17.11859

[7] Findeisen M , Allen TL , Henstridge DC , Kammoun H , Brandon AE , Baggio LL , Watt KI , Pal M , Cron L , Estevez E *et al*. (2019) Treatment of type 2 diabetes with the designer cytokine IC7Fc. Nature 574, 63–68. 31554967 10.1038/s41586-019-1601-9

[8] Cook SA (2023) Understanding interleukin 11 as a disease gene and therapeutic target. Biochem J 480, 1987–2008. 38054591 10.1042/BCJ20220160 PMC10754292

[9] McFarland‐Mancini MM , Funk HM , Paluch AM , Zhou M , Giridhar PV , Mercer CA , Kozma SC & Drew AF (2010) Differences in wound healing in mice with deficiency of IL‐6 versus IL‐6 receptor. J Immunol 184, 7219–7228. 20483735 10.4049/jimmunol.0901929

[10] Widjaja AA , Lim WW , Viswanathan S , Chothani S , Corden B , Dasan CM , Goh JWT , Lim R , Singh BK , Tan J *et al*. (2024) Inhibition of IL‐11 signalling extends mammalian healthspan and lifespan. Nature 632, 157–165. 39020175 10.1038/s41586-024-07701-9 PMC11291288

[11] Ng B , Widjaja AA , Viswanathan S , Dong J , Chothani SP , Lim S , Shekeran SG , Tan J , McGregor NE , Walker EC *et al*. (2021) Similarities and differences between IL11 and IL11RA1 knockout mice for lung fibro‐inflammation, fertility and craniosynostosis. Sci Rep 11, 14088. 34239012 10.1038/s41598-021-93623-9 PMC8266813

[12] Hunter CA & Jones SA (2015) IL‐6 as a keystone cytokine in health and disease. Nat Immunol 16, 448–457. 25898198 10.1038/ni.3153

[13] Becker M , Joseph SS , Garcia‐Carrizo F , Tom RZ , Opaleva D , Serr I , Tschop MH , Schulz TJ , Hofmann SM & Daniel C (2023) Regulatory T cells require IL6 receptor alpha signaling to control skeletal muscle function and regeneration. Cell Metab 35, 1736–1751.e7. 37734370 10.1016/j.cmet.2023.08.010 PMC10563138

[14] Sims NA , Jenkins BJ , Nakamura A , Quinn JM , Li R , Gillespie MT , Ernst M , Robb L & Martin TJ (2005) Interleukin‐11 receptor signaling is required for normal bone remodeling. J Bone Miner Res 20, 1093–1102. 15940362 10.1359/JBMR.050209

[15] Jones SA & Jenkins BJ (2018) Recent insights into targeting the IL‐6 cytokine family in inflammatory diseases and cancer. Nat Rev Immunol 18, 773–789. 30254251 10.1038/s41577-018-0066-7

